# Phytochemical Composition and Biological Activities of Extracts from Early, Mature, and Germinated Somatic Embryos of *Cotyledon orbiculata* L.

**DOI:** 10.3390/plants12051065

**Published:** 2023-02-27

**Authors:** Gokhan Zengin, Zoltán Cziáky, József Jekő, Kyung Won Kang, José Manuel Lorenzo, Iyyakkannu Sivanesan

**Affiliations:** 1 Department of Biology, Faculty of Science, Selcuk University, 42130 Konya, Turkey; 2 Agricultural and Molecular Research and Service Institute, University of Nyíregyháza, 4400 Nyíregyháza, Hungary; 3 Babo Orchid Farm, Namyangju-si 472-831, Republic of Korea; 4 Centro Tecnológico de la Carne de Galicia, Rúa Galicia Nº 4, Parque Tecnológico de Galicia, San Cibrao das Viñas, 32900 Ourense, Spain; 5 Facultade de Ciencias, Universidade de Vigo, Área de Tecnoloxía dos Alimentos, 32004 Ourense, Spain; 6 Department of Bioresources and Food Science, Institute of Natural Science and Agriculture, Konkuk University, Seoul 05029, Republic of Korea

**Keywords:** somatic embryogenesis, plant growth regulators, secondary metabolites, liquid chromatography-mass spectrometry, antioxidant activity, enzyme inhibition

## Abstract

*Cotyledon orbiculata* L. (Crassulaceae)—round-leafed navelwort—is used worldwide as a potted ornamental plant, and it is also used in South African traditional medicine. The current work aims to assess the influence of plant growth regulators (PGR) on somatic embryogenesis (SE) in *C. orbiculata*; compare the metabolite profile in early, mature, and germinated somatic embryos (SoEs) by utilizing ultra-high performance liquid chromatography-tandem mass spectrometry (UHPLC-MS/MS); and determine the antioxidant and enzyme inhibitory potentials of SoEs. A maximum SoE induction rate of 97.2% and a mean number of SoEs per *C. orbiculata* leaf explant of 35.8 were achieved on Murashige and Skoog (MS) medium with 25 µM 2,4-Dichlorophenoxyacetic acid and 2.2 µM 1-phenyl-3-(1,2,3,-thiadiazol-5-yl)urea. The globular SoEs were found to mature and germinate best on MS medium with gibberellic acid (4 µM). The germinated SoE extract had the highest amounts of both total phenolics (32.90 mg gallic acid equivalent/g extract) and flavonoids (1.45 mg rutin equivalent/g extract). Phytochemical evaluation of SoE extracts by UHPLC-MS/MS reveals the presence of three new compounds in mature and germinated SoEs. Among the SoE extracts tested, germinated SoE extract exhibited the most potent antioxidant activity, followed by early and mature somatic embryos. The mature SoE extract showed the best acetylcholinesterase inhibitory activity. The SE protocol established for *C. orbiculata* can be used for the production of biologically active compounds, mass multiplication, and conservation of this important species.

## 1. Introduction

*Cotyledon orbiculata* L.—a member of the Crassulaceae—is commonly called round-leafed navelwort or pig’s ear, is native to South Africa, and is typically found in Southern Africa [1]. *C. orbiculata* is widely used as a potted plant worldwide due to its attractive bellflowers along with its leaves and low-care requirements. In South African traditional medicine, leaves collected from the wild populations of *C. orbiculata* are used to treat deworming, earache, inflammation, neurological problem, skin infection, and wounds [2,3]. The crude extracts obtained from the aerial parts of *C. orbiculata* have been shown to possess anticancer [4], anticonvulsant [1], anti-inflammatory [5,6], antimicrobial [2,7], antinociceptive [5], antioxidant [6], and anthelmintic [2,8] activities. Several bufadienolides, including cotyledosides [9], orbicusides, and tyledoside C [10], are found in the aerial parts of *C. orbiculata*. Phytochemical analysis of *C. orbiculata* leaf extract has also confirmed the presence of cardiac glycosides, flavonoids, phenolics, reducing sugars, saponins, condensed tannin, gallotannin, and triterpene steroids [1,2,3]. Due to its ornamental and medicinal values, wild populations of *C. orbiculata* are collected extensively; therefore, it has been designated as a near-threatened plant in parts of South Africa [7,11]. The traditional propagation methods are Inefficient in their ability to meet the current demand for *C. orbiculata* due to the shortage of planting materials. Hence, an efficient method for propagating *C. orbiculata* is needed to achieve its mass production and germplasm conservation.

Micropropagation is an in vitro culture method that is widely used for the mass propagation of various plants [12,13]. Somatic embryogenesis (SE) is one of the most efficient micropropagation methods [14], and it is widely used for mass propagation [15,16], virus elimination [17], germplasm conservation [18], genetic transformation [19], synthetic seeds [20], and secondary metabolites [21] production. SE is the developmental process of somatic cell differentiation into a somatic embryo (SoE) [22]. Several factors, including culture medium composition [23], explant type [24], plant growth regulators [25] (PGR), and culture environment [26], affect the formation of somatic embryo. PGR play a vital role in the induction, development, and conversion of somatic embryos [25,26]. Research has shown that the addition of PGR is required for the induction of somatic embryos in vitro in Crassulaceae members such as *Crassula ovata* [27], *Kalanchoe blossfeldiana* [28], and *Orostachys japonicus* [29]. To date, there has been no report investigating the somatic embryogenesis of *Cotyledon* species.

Kumari et al. [7] reported an in vitro method for *C. orbiculata* regeneration via organogenesis. The authors also showed that ethanolic extracts from calli, in vitro-raised shoots and plantlets, and leaves of ex vitro-grown *C. orbiculata* (2-month-old) had higher antimicrobial activity against *Klebsiella pneumoniae* than mother plants (10-year-old) leaves extract. However, they did not examine the bioactive metabolites in the tissues of *C. orbiculata*. Further, there has been no study examining the production of bioactive compounds from in vitro cultures of *Cotyledon* species. Several studies have confirmed the presence of diverse phytochemicals in *C. orbiculata* extracts [1,2,3]. Still, the phytochemical profile of *C. orbiculata* has not been documented, except for bufadienolides. Liquid chromatography with tandem mass spectrometry (LC-MS/MS) is the most effective method for the qualitative detection and identification of major and minor compounds in plant extracts [30,31].

This work aims to assess the impact of PGR on somatic embryogenesis in *C. orbiculata*; compare the metabolite profile in early, mature, and germinated somatic embryos by utilizing UHPLC-MS/MS; and determine the antioxidant and enzyme inhibitory potential of somatic embryos.

## 2. Materials and Methods

### 2.1. Somatic Embryogenesis (SE)

Healthy, young shoots isolated from greenhouse-raised *Cotyledon orbiculata* (L.) plants were soaked in a mild detergent solution and kept under running tap water for 30 min. Shoots were disinfected in ethanol (70%, 90 s), then mercuric chloride (0.1%, 15 min), followed by three washes (60 s per wash) in sterilized distilled water and air-dried. Leaves were dissected, cut into 0.6–1.0 cm long segments, and placed in a sterilized culture bottle (500 mL) containing Murashige and Skoog [32] (MS) medium with 8 g/L agar, 30 g/L sucrose, and 0–30 µM of 2,4-Dichlorophenoxyacetic acid (2,4-D), along with indole-3-acetic-acid (IAA), indole-3-butyric acid (IBA), and α-naphthalene acetic acid (NAA) or 1.2–8.8 µM of N^6^-benzyladenine (6-BA), kinetin (KN) and 1-phenyl-3-(1,2,3,-thiadiazol-5-yl)urea (TDZ) plus 25 µM of 2,4-D for SoE induction. The pH of the SoE medium was adjusted to 5.7 and autoclaved for 22 min at 122 °C. The culture bottles were incubated in the darkness for three weeks, then kept under a 16-h photoperiod (40–45 µMol s^−1^ m^−2^) for nine weeks at a temperature of 23 to 26 °C. Fifty leaf segments were used per treatment, with three repetitions. The leaf segments were assessed for SoE induction after 12 weeks. The SoE induction percentage was calculated as the number of leaf segments with SoEs divided by the total number of leaf segments cultured × 100 [33]. Globular SoEs were subcultured into the MS medium with 0, 1, 2, 4, or 8 µM 6-BA or gibberellic acid (GA_3_), then proceeded to further development and germination. The cultures were kept under a 16-h photoperiod (20–25 µMol s^−1^ m^−2^) at temperatures from 23 to 26 °C. Fifty globular SoEs were used per treatment, with three repetitions. After eight weeks, the SoE conversion percentage was calculated as the number of germinated SoEs divided by the total number of SoEs cultured × 100 [34].

### 2.2. Phytochemical Analysis

#### 2.2.1. Extract Preparation

Early (globular), mature (torpedo), and germinated (cotyledonary) SoEs were lyophilized. The extracts were obtained using a homogenizer-assisted extraction. In the procedure, *C. orbiculata* samples (50 mg) were extracted with 80% methanol using an Ultraturrax at 6000 g for 30 min. After filtration, the extracts were dried using a rotary vacuum evaporator before being stored at 4 °C until further analysis.

#### 2.2.2. Estimation of Total Phenolics Content (TPC) and Flavonoids Content (TFC)

The TPCs of *C. orbiculata* SoEs extracts were determined using the Folin–Ciocalteu reagent described by Slinkard and Singleton [35], and the results were expressed in terms of mg of gallic acid equivalent (GAE). The TFCs of *C. orbiculata* SoEs extracts were determined using the aluminum chloride (AlCl_3_) method described by Zengin et al. [36] and calculated in terms of mg of rutin equivalent (RE).

#### 2.2.3. Chemical Characterization

A previously optimized and described UHPLC/MS/MS technique was used to screen the chemical compositions of three extracts containing phenolic and flavonoid compounds. Mass spectrometry was conducted using an electrospray ionization source (ESI) operating in both negative and positive ion modes. Mass spectra were recorded as full MS between *m/z* 100 and 1500 atomic mass units and MS/MS mode using a Q-Exactive (Thermo Fisher Scientific) Orbitrap mass spectrometer. These data can be examined to detect and confirm analytes in complex matrices. The detected compounds were identified through comparison with authentic standards, their MS/MS spectra and fragmentation patterns, and their HRMS spectral information. All data were processed using the TraceFinder software and tentatively identified by comparing their retention time (*R*_t_) and mass spectrum with the reported data and our spectral library. The difference between the mass of measured and calculated* exact protonated or deprotonated molecular ions was less than 5 ppm [37]. 

### 2.3. Biological Activities of C. orbiculata SoEs Extracts

#### 2.3.1. Antioxidant Assay

The antioxidant capacity of *C. orbiculata* SoEs extracts was estimated using the metal chelating ability (MCA), phosphomolybdenum (total antioxidant capacity, PBD), ferric reducing antioxidant power (FRAP), cupric reducing antioxidant capacity (CUPRAC), 2,2-diphenyl-1-picrylhydrazyl (DPPH), and 2,2-azino-bis (3-ethylbenzothiazoline-6-sulphonic acid) (ABTS) methods described by Uysal et al. [38]. Assays were performed in triplicate. The results are presented as IC_50_ values (mg/mL). 

#### 2.3.2. Enzyme Inhibition Assay

The amylase, acetylcholinesterase (AChE), tyrosinase, and butyrylcholinesterase (BChE) inhibitory effects of *C. orbiculata* SoEs extracts were each conducted in triplicate according to the procedures described by Uysal et al. [38]. The results are given as IC_50_ values (mg/mL).

### 2.4. Statistical Analysis

Data were subjected to analysis of variance (ANOVA), and significant differences (*p* < 0.05) among means were determined by Duncan’s multiple range test (DMRT) using SAS version 9.4 (SAS Institute, Cary, NC, USA).

## 3. Results

### 3.1. Somatic Embryogenesis (SE)

#### 3.1.1. Influence of Auxins on SE in *C. orbiculata*

**Figure 1:**
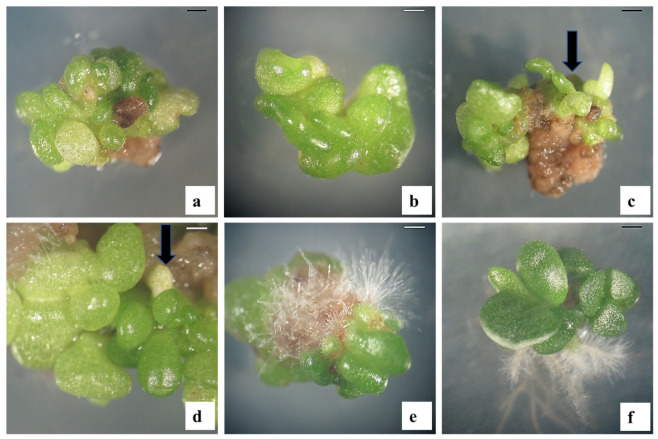
SE and plant regeneration from leaf explants of *C. orbiculata*: (**a**) initiation of globular-shaped SoE on MS medium with 25 µM 2,4-D (8 weeks); (**b**) formation of heart-shaped SoE on MS medium with 25 µM 2,4-D and 1.2 µM TDZ (10 weeks); (**c**) formation of the torpedo (arrow) shaped SoE on MS medium with 25 µM 2,4-D and 1.2 µM TDZ (12 weeks); (**d**) formation of cotyledonary (arrow) shaped SoE on MS medium with 2 µM GA_3_ (5 weeks); (**e**) germination of SoE (8 weeks); (**f**) *C. orbiculata* plantlets from SoE (10 weeks). Scale bar. (**a**–**f**) 1.0 mm.

The surface sterilization of *C. orbiculata* shoots with ethanol and mercuric chloride resulted in 100% sterile leaf culture. The explants cultivated on MS PGR-free medium (control) did not produce SoEs or callus. On the other hand, the explants of *C. orbiculata* developed callus, root, or SoE within 45 days of culture on an auxin-containing medium. However, the addition of auxin at 5 or 10 µM in the cultivation medium did not support SE. The SoE formation occurred at the cut edges of *C. orbiculata* leaf segments in the presence of 15–30 µM auxin (Table 1). After eight weeks of cultivation, pale green globular SoEs were detected (Figure 1a). The ANOVA showed that auxin type, auxin concentration, and the interaction of type and concentration of auxin all had significant (*p* < 0.001) effects on SoE induction and the number of SoE developed per explant (Table 1). Of the studied auxin types, a high rate of SoE induction was obtained on 2,4-D (25.6%), followed in descending order by NAA (16.9%), IBA (13.9%), and IAA (11.7%). Similarly, 2,4-D produced the highest mean number of SoEs (6.3), followed in descending order by NAA (4.1), IBA (2.6), and IAA (2.2). Of the studied auxin concentrations, a high incidence of SoE induction was obtained on 25 µM (34.7%), followed in descending order by 20 µM (31.3%), 30 µM (23.9%), and 15 µM (13.4%). Lastly, 25 µM produced the highest mean number of SoEs (8.0), followed in descending order by 20 µM (6.7), 30 µM (4.7), and 15 µM (3.4). The maximum SoE induction rate (60.6%) and mean number of SoEs per *C. orbiculata* leaf explant (14.9) were achieved on an MS medium with 25 µM of 2,4-D (Table 1). Thus, 25 µM of 2,4-D was selected for the additional SE experiments.

#### 3.1.2. Effect of Cytokinins Plus 25 µM 2,4-D on SE in *C. orbiculata*

The addition of cytokinins to the 2,4-D (25 µM) containing MS medium significantly (*p* < 0.05) affected the rate of SoE induction and the mean number of SoEs. Different developmental stages (globular, heart, and cotyledonary) of SoEs were observed from *C. orbiculata* leaf explants within 12 weeks of culturing on MS medium with 25 µM 2,4-D and cytokinins (Figure 1b,c). The rate of SoE induction and the number of SoEs were both significantly (*p* < 0.001) affected by cytokinin type, cytokinin concentration, and their interaction (Table 2). Of the tested cytokinin types, a high rate of SoE induction was obtained on TDZ (77.3%), followed in descending order by KN (75.1%), and 6-BA (70.6%). Similarly, TDZ produced the highest mean number of SoEs (24.9), followed in descending order by KN (18.4) and 6-BA (14.7). Among the studied cytokinin concentrations, a high incidence of SoE induction was obtained on 4.4 µM (85.9%) followed in descending order by 2.2 µM (83.8%), 1.2 µM (76.4%), and 8.8 µM (51.3%). By contrast, 2.2 µM produced the highest mean number of SoEs (25.5), followed in descending order by 4.4 µM (23.0), 1.2 µM (19.0), and 8.8 µM (9.7).

The optimal SE medium (MS + 25 µM 2,4-D), with the addition of 4.4 µM 6-BA, led to the maximum rate of SoE induction (88.7%) and number of SoEs (21.3). Increasing the 6-BA dose from 1.2 to 4.4 µM in the optimal SE medium led to an increase in the rate of SoE induction from 66.6% to 88.7% and an increase in the mean number of SoEs from 11.8 to 21.3. However, an increase in the 6-BA dose beyond 4.4 µM reduced the frequency of SoE induction (52.6%) and the average number of SoEs (7.4). When the optimal SE medium was combined with KN (1.2–8.8 µM), 56.9–92.3% of *C. orbiculata* leaf explants produced a mean of 8.0–27.0 SoEs. The optimal SE medium supplemented with 4.4 µM KN was found to be the best in SoE production from *C. orbiculata* leaf explants (Table 2). Adding TDZ (1.2–4.4 µM) to optimal SE medium improved production SoEs. The greatest rate of SoE induction (97.2%) and the highest mean number of SoEs (35.8) were obtained with optimal SE medium with 2.2 µM TDZ (Table 2). Raising the TDZ doses above 2.2 µM reduced the SE response of *C. orbiculata* leaf explants.

#### 3.1.3. Effect of 6-BA and GA_3_ on Conversion of *C. orbiculata* SoEs

**Figure 2:**
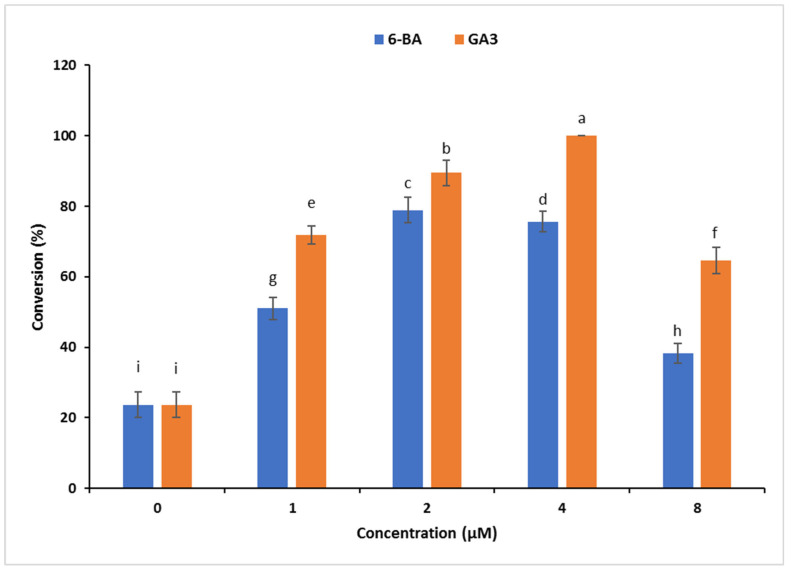
Effect of 6-BA and GA_3_ on the conversion of *C. orbiculata* SoEs. The means ± SDs in each vertical bar with different alphabets (a–i) are significantly different according to DMRT at *p* < 0.05.

Within eight weeks, globular SoEs matured and germinated. Only a few SoEs germinated on the control (MS) medium. The conversion of globular SoEs was boosted by supplementing MS medium with 6-BA and GA_3_ (1–8 µM). The frequency of SoE conversion ranged from 23.7% to 100%. Among the two tested PGRs, GA_3_ proved to be the most effective for converting *C. orbiculata* SoEs. The highest rate of SoE conversion (100%) was attained on a medium with 4 µM GA_3_ (Figure 2).

### 3.2. Phytochemical Analysis 

In the present study, we determined the total amounts of phenolic and flavonoids of *C. orbiculata* extracts. The results are presented in Table 3. Among the tested samples, the highest levels of total phenolics and flavonoids were determined in the extract of germinated somatic embryos (32.90 mg GAE/g extract and 1.45 mg RE/g extract, respectively). The extracts from early and mature somatic embryos contained almost the same contents of total phenolics and flavonoids.

**Figure 3:**
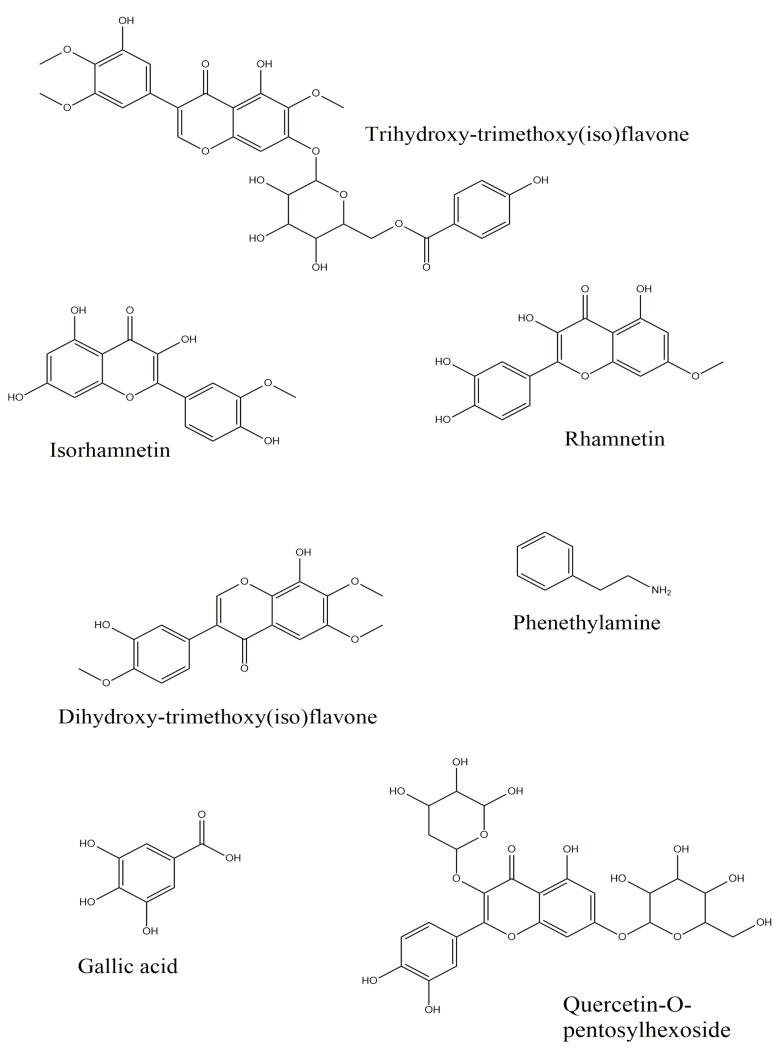
Structures of some compounds in the tested extracts.

The characterized compounds are listed in Table 4. The chromatographic and mass spectrometric data (retention times, protonated or deprotonated molecular ions, fragment ions) and assigned identities for compounds were given Tables S1–S3. In total, 38 compounds were identified in the extracts. A total of 32 compounds were found in early somatic embryo extract, 33 were found in mature somatic embryo extract, and 32 were found in germinated somatic embryo extract (Figures S1–S3). The structures of some compounds are given in Figure 3.

The samples showed similar chromatographic profiles, and a wide range of compounds—mainly derivatives of nicotinic acid and flavones—were characterized. The lowest molecular mass component was nicotinamide (**3**) (rt: 1.61–1.63 min, [M+H]^+^: 123.0559*), and two compounds had the highest molecular mass. These were characterized as Hyperoside (**15**) (rt: 23.22 min, [M−H]^−^: 463.0877*) and Isoquercitrin (**16**) (rt: 23.44 min, [M−H]^−^: 463.0877*). The positive ion mode of ESI-MS/MS was a powerful complementary tool of the negative ion mode for the determination of the chemical structure of the compounds. In many cases, the sensitivity was higher and more fragment ions could be detected in positive mode; examples include Nicotinic acid and its derivatives, oxybutanedioic acid derivatives, hydroxy-, dihydroxy- and trihydroxy-methoxy/dimethyoxy/trimethoxy(iso)flavones. The major advantage of negative ion mode (ESI^−^) is the reduced background noise.

The exact identification of constitutional isomers detected in extracts is not possible even when using high mass resolution MS measurements, for example, Luteolin-O-hexoside isomers, Trihydroxy-trimethoxy(iso)flavone, Dihydroxy-trimethoxy(iso)flavone isomers, Dihydroxy-dimethoxy(iso)flavone, Dimethoxy(iso)flavone, Trimethoxy(iso)flavone, Dihydroxy-methoxy(iso)flavone, Hydroxy-trimethoxy(iso)flavone, and Hydroxy-methoxy(iso)flavone. 

### 3.3. Antioxidant Abilities

We determined the antioxidant properties of *C. orbiculata* extracts, and the results are presented in Table 5. Among the antioxidant assays, DPPH and ABTS are the most popular for evaluating plant extracts’ radical scavenging ability. As can be seen in Table 5, the most active extract was germinated somatic embryos with an IC_50_ of 0.62 mg/mL, followed by early and mature somatic embryos. However, Trolox showed a stronger ability to scavenge free radicals compared to the tested extracts. The transformations of cupric to cuprous and ferric to ferrous reflect the electron-donating ability of antioxidant compounds, and the mechanism is known to be the reduction in power. For this purpose, we performed CUPRAC and FRAP assays. In both assays, the best reduction ability was provided by germinated somatic embryos (CUPRAC: 0.92 mg/mL; FRAP: 0.55 mg/mL). However, all extracts were less active than the standard antioxidant, Trolox. Phosphomolybdenum (PBD) assay is one of the total antioxidant assays, and all antioxidant compounds could play an effective role in the assay. As presented in Table 5, the tested samples were in descending order of germinated > early >mature. The chelation of transition metals can hinder the production of hydroxyl radicals via the Fenton reaction and, therefore, be considered an important antioxidant mechanism. In contrast to other assays, the extracts of early and germinated somatic embryos exhibited similar chelating abilities. However, the extract of mature somatic embryos showed the weakest chelating ability. Moreover, EDTA was shown to be an excellent chelator with the lowest IC_50_ value (0.02 mg/mL).

### 3.4. Enzyme Inhibition Effects

The present study reported the enzyme inhibitory properties of *C. orbiculata* extracts against AChE, BChE, tyrosinase, and amylase. The results are listed in Table 6. In the AChE inhibition assay, the mature somatic embryo extract provided the best inhibition with the lowest IC50 value (0.75 mg/mL). The early and germinated somatic embryo extracts had almost the same inhibitory potency. Regarding the BChE inhibition assay, the best effect was found in the germinated somatic embryo extract, but the ability was close to that of the mature somatic embryo extract. The extract of early somatic embryos was found to have the weakest ability to inhibit BChE. Tyrosinase is a key enzyme in melanogenesis, and its inhibition is important for controlling hyperpigmentation problems. As listed in Table 6, the tested extracts showed similar tyrosinase inhibitory activities, and the most active one was from the germinated somatic embryos. However, kojic acid was the superior inhibitor with the lowest IC50 (0.08 mg/mL). Amylase is the main enzyme involved in the hydrolysis of carbohydrates, and its inhibition can control blood sugar levels in diabetics. The highest amylase inhibition was achieved by early somatic embryos, followed by germinated and mature somatic embryos. All extracts also showed weaker abilities compared to acarbose (IC_50_: 0.68 mg/mL).

## 4. Discussion

The surface sterilization of explants (plant materials) is an essential aspect of establishing in vitro aseptic culture [39]. In this study, disinfection of *C. orbiculata* shoots resulted in a 100% sterile in vitro culture. A similar disinfection method was also used to obtain sterile explants of *C. orbiculata* [7]. The control (MS) medium and MS medium supplemented with lower levels (5 and 10 µM) of auxin failed to promote SE in *C. orbiculata*. However, incorporating high levels (above 10 µM) of auxin resulted in SoE induction from leaf explants of *C. orbiculata* (Table 1). In many species, the presence of auxin—often at high concentrations—is required to induce SoEs [26,40,41]. In this study, 2,4-D (25 µM) proved to be significantly (*p* < 0.001) superior in inducing SE from leaf explants of *C. orbiculata* than NAA, IBA, and IAA, which is likely attributable to the fact that the degradation rate of 2,4-D is lower than those of other studied auxins. The effectiveness of 2,4-D for stimulating SE has already been disclosed in various species [24,26,33,40,41,42]. The 2,4-D and cytokinin combination was frequently used to enhance SoE induction in most species. The addition of cytokinin (6-BA, KN, or TDZ at 1.2–4.4 µM) to the SE-promoting level (25 µM) of 2,4-D significantly enhanced the formation of SoEs (Table 2). The combination of 2,4-D and 6-BA has been shown to be effective for the induction of SoEs in *Ananas comosus* [15], *Betula platyphalla* [43], *Campanula punctata* [24], *Crassula ovata* [27], *Orostachys japonicus* [29], and *Picea pungens* [44]. Similarly, a combination of 2,4-D and KN was effective for the induction of SoEs in chrysanthemum ‘Hornbill Dark’ [45], *Trachyspermum ammi* [46], and *Viola canescens* [47]. Likewise, a combination of 2,4-D and TDZ was found to be the best for the induction of SoEs in *Camellia oleifera* [48], *Hippeastrum* [49], *Prunus dulcis* [50], and *Tulipa gesneriana* [51]. Among the texted cytokinins, the highest rate of SoE induction with the maximum number of SoEs per *C. orbiculata* leaf explant was achieved using the optimal SE medium with TDZ (Table 2).

TDZ is a PGR that is often used for the induction of SoEs and callus, adventitious shoot regeneration, and multiple shoot induction in various plants [52]. It is often combined with other PGRs to achieve the best in vitro culture results. However, the ratio of auxin and cytokinin significantly affects SE. In this study, the best rate of SoE formation (97.2%) with a maximum number of SoEs per *C. orbiculata* leaf explant (35.8) was obtained in the MS medium containing 2,4-D (25 µM) and TDZ (2.2 µM). Similarly, the presence of a high level of auxin (22.5 µM of 2,4-D) and a low level of cytokinin (2.2 µM of 6-BA) was found to be effective for SE in *Orostachys japonicus* [29]. By contrast, a low level of auxin (2.3 µM of 2,4-D) and a high level of cytokinin (4.4 µM of 6-BA) were found to be the best conditions for SE in *Crassula ovata* [27]. Therefore, the requirement of the PGRs ratio for SE in Crassulaceae varies according to genus. The globular-, heart-, and torpedo-stage SoEs were formed when the *C. orbiculata* leaf explants were cultured on optimal SE induction medium with TDZ (Figure 1a–c). However, only a few globular SoEs matured, and germination was not accomplished. Similar results have also been reported in another Crassulaceae member, *Orostachys japonicus* [29]. SoE maturation and subsequent plantlet conversion are often affected by the presence of PGRs in the SE medium. Globular SoE conversion (maturation and germination) has commonly been achieved on PGR-free medium [22,41]; however, in some species, the addition of cytokinins [21,29,49], abscisic acid [44,53], or GA_3_ [33,34] is needed for the development and germination of SoE. In this study, the highest conversion of *C. orbiculata* SoE was accomplished on a medium with 4 µM GA_3_. GA_3_ has been reported to have positive effects on SoE conversion in *Haworthia retusa* [33], *Hosta minor* [34], and *Juglans regia* [54].

**Figure 4:**
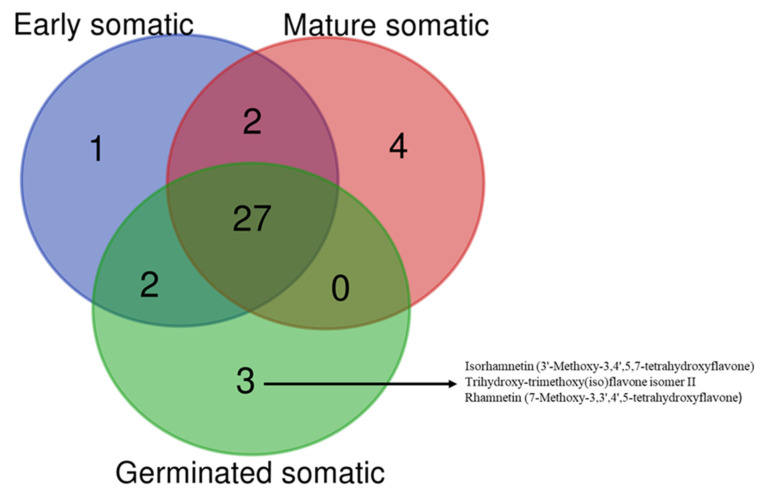
Venn diagram showing the number of compounds identified in the (SoE) tested extracts.

Over the last decade, phenols have attracted more interest in nutraceutical and pharmaceutical applications due to their promising biological activities [55]. In this sense, when the content of phenols in an extract is detected, it is a significant indicator of its biological effects. In the current work, the extract of germinated somatic embryos was found to have the highest total phenolic and flavonoid content. In a previous study conducted by Ondua et al. [6], the total phenolic level of *C. orbiculata* extracts varied from 1.34 (in n-hexane extract) to 23.93 mg GAE/g (in methanol extract), which is lower than that of the extract from germinated somatic embryos tested in the study. Although the spectrophotometric methods could provide initial insight into the pharmacological value of plant extracts, certain concerns have recently arisen from the assays. Due to the complex nature of plant extracts, not only will certain compounds of interest react with the reagent used in the assays, but so will other phytochemicals. Therefore, the results of these assays could be suspect. Keeping this in mind, chromatographic techniques are needed to obtain more accurate chemical profiles of plant extracts. In the present study, the chemical composition of the tested extracts was characterized using the UHPLC/MS/MS technique, and the results are listed in Table 2. The extracts had a similar chemical composition, and, interestingly, new compounds were identified in mature SoE (33–35) and germinated SoE (36–38) (Figure 4).

Over the last century, most people have come to be familiar with the term antioxidant. The term denotes protection against free radical attacks that affect the progression of serious health problems such as cancer, diabetes, or obesity. Several studies have reported that antioxidant intake is inversely associated with the development of these diseases [56,57]. With this in mind, we determined the antioxidant properties of *C. orbiculata* extracts, and the results are presented in Table 5. The germinated somatic embryo extract generally showed stronger antioxidant ability than other tested extracts. Ondua et al. [6] reported that the IC_50_ values of the methanol extract of *C. orbiculata* were 3.76 g/mL and 3.35 g/mL for DPPH and ABTS, respectively. Based on their results, our extracts showed weaker free radical scavenging ability than their tested extracts. From Table 5, when the combined scavenger and reduction performance results were obtained, we found an almost similar order. The obtained results almost agreed with the results of the total phenolic and flavonoid content of the tested extracts; therefore, we concluded that phenolics made the main contribution to the radical scavenging and reducing ability. Moreover, some compounds have only been detected in germinated SoE extract, and these are also known to be powerful antioxidants [58,59].

Enzymes are pharmaceutical targets for treating various health problems, including Alzheimer’s disease, obesity and diabetes. In particular, the inhibition of key enzyme abilities might alleviate the symptoms of the diseases mentioned above [60]. For this purpose, several compounds have been manufactured as enzyme inhibitors, and most of them are presented on pharmacy shelves. However, synthetic compounds exhibit unpleasant side effects, including gastrointestinal problems and toxicity [61,62,63]. Therefore, several studies have focused on replacing synthetic inhibitors with natural ones. The tested extracts showed remarkable inhibitory effects on AChE, BChE, tyrosinase, and amylase. The observed capabilities of the tested samples can be explained by the presence of some chemical compounds. As listed in Table 6, some compounds have been reported to serve as enzyme inhibitors [64,65,66]. The current work is the first report examining the enzyme-inhibitory effect of *C. orbiculata*. Thus, these results could establish future directions for studies using *C. orbiculata* to develop functional applications.

## 5. Conclusions

In this work, direct SE from the leaf tissue was described for the first time. Among the studied auxin types, the highest rate of SoE induction was obtained on 2,4-D, followed in descending order by NAA, IBA, and IAA. The inclusion of cytokinin (6-BA, KN, or TDZ at 1.2–4.4 µM) in the optimal SE medium MS containing 25 µM of 2,4-D enhanced the formation of SoEs. In total, 38 metabolites were identified in *C. orbiculata* SoEs by UHPLC-MS/MS. Among them, quercetin-O-pentosylhexoside, dihydroxy-trimethoxy(iso)flavone isomer 1, dihydroxy-trimethoxy(iso)flavone isomer 2 (33–35), isorhamnetin (3′-Methoxy-3,4′,5,7-tetrahydroxyflavone), and rhamnetin (7-Methoxy-3,3′,4′,5-tetrahydroxyflavone, trihydroxy-trimethoxy(iso)flavone) isomer II (36–38) are only found in mature SoEs and germinated SoEs, respectively.

## Figures and Tables

**Table 1:** Impact of auxins on SE in *C. orbiculata*.

Auxin	Auxin Conc. (µM)	SoE Induction (%)		Number of SoEs per Explant	
Control	0	0.0 ± 0.0 ^k^		0.0 ± 0.0 ^j^	
2,4-D	5	0.0 ± 0.0 ^k^		0.0 ± 0.0 ^j^	
	10	0.0 ± 0.0 ^k^		0.0 ± 0.0 ^j^	
	15	23.1 ± 4.2 ^g^		5.4 ± 1.0 ^e^	
	20	32.7 ± 4.1 ^de^		9.8 ± 1.9 ^b^	
	25	60.6 ± 3.5 ^a^		14.9 ± 2.1 ^a^	
	30	43.2 ± 5.2 ^b^		7.6 ± 1.5 ^d^	
IAA	5	0.0 ± 0.0 ^k^		0.0 ± 0.0 ^j^	
	10	0.0 ± 0.0 ^k^		0.0 ± 0.0 ^j^	
	15	10.7 ± 2.9 ^i^		2.9 ± 0.8 ^h^	
	20	34.2 ± 4.5 ^d^		5.3 ± 1.0 ^e^	
	25	20.2 ± 4.7 ^h^		3.6 ± 1.3 ^gh^	
	30	4.9 ± 1.3 ^j^		1.7 ± 0.7 i	
IBA	5	0.0 ± 0.0 ^k^		0.0 ± 0.0 ^j^	
	10	0.0 ± 0.0 ^k^		0.0 ± 0.0 ^j^	
	15	7.1 ± 1.5 ^j^		1.9 ± 0.8 ^i^	
	20	18.7 ± 2.9 ^h^		2.9 ± 1.1 h	
	25	30.6 ± 3.2 ^e^		6.7 ± 1.6 ^d^	
	30	24.0 ± 3.5 ^g^		4.3 ± 1.0 ^fg^	
NAA	5	0.0 ± 0.0 ^k^		0.0 ± 0.0 ^j^	
	10	0.0 ± 0.0 ^k^		0.0 ± 0.0 ^j^	
	15	12.7 ± 2.5 ^i^		3.6 ± 0.9 ^gh^	
	20	39.6 ± 2.5 ^c^		8.7 ± 1.0 c	
	25	27.6 ± 4.6 ^f^		7.0 ± 1.1 ^d^	
	30	21.4 ± 3.1 ^gh^		5.1 ± 1.1 ^ef^	
ANOVA	R-square	0.9738		0.9411	
	Coefficient of variation	16.93		26.58	
	Root mean square error	2.89		1.01	
		F-value	*p*-value	F-value	*p*-value
	Auxin type	285.68	0.001	176.20	0.001
	Auxin conc.	984.44	0.001	394.14	0.001
	Auxin type *Auxin conc.	90.29	0.001	37.81	0.001

Means ± standard deviations (SDs) within columns (3 and 4) followed by different alphabets (^a–k^) are significantly different according to DMRT at *p* < 0.05. *—Interaction.

**Table 2:** Effect of combinations of 25 µM 2,4-D and cytokinins on SE in *C. orbiculata*.

Cytokinin Type	Cytokinin Conc. (µM)	SoE Induction (%)		Number of SoEs per Explant	
Control (25 µM 2,4-D)	0	60.6 ± 3.5 ^h^		14.9 ± 2.1 ^gf^	
6-BA	1.2	66.6 ± 2.7 ^g^		11.8 ± 1.4 ^g^	
	2.2	74.8 ± 3.0 ^ef^		18.1 ± 2.9 ^e^	
	4.4	88.7 ± 4.6 ^c^		21.3 ± 2.7 ^d^	
	8.8	52.6 ± 5.8 ^j^		7.4 ± 1.2 ^h^	
KN	1.2	72.0 ± 3.9 ^f^		15.9 ± 2.2 ^f^	
	2.2	79.3 ± 3.1 ^d^		22.6 ± 1.8 ^d^	
	4.4	92.3 ± 3.0 ^b^		27.0 ± 2.9 ^c^	
	8.8	56.9 ± 3.1 ^i^		8.0 ± 2.2 ^h^	
TDZ	1.2	90.6 ± 2.7 ^bc^		29.3 ± 2.9 ^b^	
	2.2	97.2 ± 2.8 ^a^		35.8 ± 2.5 ^a^	
	4.4	76.9 ± 4.3 ^de^		20.8 ± 1.9 ^d^	
	8.8	44.6 ± 3.4 ^k^		13.6 ± 2.2 ^g^	
ANOVA	R-square	0.9561		0.9356	
	Coefficient of variation	4.89		11.91	
	Root mean square error	3.64		2.29	
		F-value	*p*-value	F-value	*p*-value
	Cytokinin type	31.38	0.001	181.54	0.001
	Cytokinin conc.	513.87	0.001	247.16	0.001
	Cytokinin type * Cytokinin conc.	80.74	0.001	48.55	0.001

Means ± SDs within columns (3 and 4) followed by different alphabets (^a–k^) are significantly different according to DMRT at *p* < 0.05. *—Interaction.

**Table 3:** Total phenolic and flavonoid contents of the tested samples.

Samples	Total Phenolic Content (mg GAE/g)	Total Flavonoid Content (mg RE/g)
Early somatic embryos	21.28 ± 0.05 ^b^	0.97 ± 0.10 ^b^
Mature somatic embryos	21.32 ± 0.20 ^b^	0.95 ± 0.04 ^b^
Germinated somatic embryos	32.90 ± 0.46 ^a^	1.45 ± 0.04 ^a^

Values are expressed as mean ± S.D. GAE: Gallic acid equivalent; RE: rutin equivalent. Different letters indicate significant differences in the tested samples (*p* < 0.05).

**Table 4:** Chemical composition of *Cotyledon orbiculata* extracts.

Compounds	Early Somatic Embryo	Mature Somatic Embryo	Germinated Somatic Embryo
Trigonelline	+	+	+
Nicotinic acid (Niacin)	+	+	+
Nicotinamide	+	+	+
Gallic acid (3,4,5-Trihydroxybenzoic acid) ^1^	+	−	+
Phenethylamine	+	−	−
Dihydroxybenzoic acid	+	+	+
Caffeic acid	+	+	+
Taxifolin (Dihydroquercetin) ^1^	+	+	+
cis-3-[(4-hydroxy-3-Methoxyphenyl)-prop-2-enoyl]oxybutanedioic acid	+	+	+
Eriodictyol-O-hexoside	+	+	+
trans-3-[(4-hydroxy-3-methoxyphenyl) prop-2-enoyl]oxybutanedioic acid	+	+	+
Quercetin-O-pentosylhexoside	−	+	−
Luteolin-O-hexoside isomer 1	+	+	+
Luteolin-O-hexoside isomer 2	+	+	−
Hyperoside (Quercetin-3-O-galactoside)	+	+	+
Isoquercitrin (Quercetin-3-O-glucoside) ^1^	+	+	+
Eriodictyol (3′,4′,5,7-Tetrahydroxyflavanone) ^1^	+	+	+
Quercetin (3,3′,4′,5,7-Pentahydroxyflavone) ^1^	+	+	+
Naringenin (4′,5,7-Trihydroxyflavanone) ^1^	+	+	+
Luteolin (3′,4′,5,7-Tetrahydroxyflavone) ^1^	+	+	+
Chrysoeriol (3′-Methoxy-4′,5,7-trihydroxyflavone)	+	+	−
Dihydroxy-trimethoxy(iso)flavone isomer 1	−	+	−
Apigenin (4′,5,7-Trihydroxyflavone) ^1^	+	+	+
Isorhamnetin (3′-Methoxy-3,4′,5,7-tetrahydroxyflavone) ^1^	−	−	+
Trihydroxy-trimethoxy(iso)flavone isomer I	+	+	+
Rhamnetin (7-Methoxy-3,3′,4′,5-tetrahydroxyflavone)	−	−	+
Trihydroxy-trimethoxy(iso)flavone isomer II	−	−	+
Dihydroxy-dimethoxy(iso)flavone	+	+	+
Dihydroxy-trimethoxy(iso)flavone isomer 2	−	+	−
Chrysin (5,7-Dihydroxyflavone)	+	+	+
Dimethoxy(iso)flavone	+	+	+
Galangin (3,5,7-Trihydroxyflavone) ^1^	+	+	+
Trimethoxy(iso)flavone	+	+	+
Dihydroxy-methoxy(iso)flavone	+	+	+
Hydroxy-trimethoxy(iso)flavone	+	+	+
Hydroxy-methoxy(iso)flavone	+	+	+
Linoleamide	+	+	+
Oleamide	+	+	+

^1^ Confirmed by standard. −: not detected; +: detected. Aside from flavone derivatives, Linoleamide and Oleamide were also detected in the extracts. These compounds belong to the class of organic compounds known as fatty amides, and they are natural plant metabolites; another way to introduce these compounds may be as the external source in the extraction process.

**Table 5:** Antioxidant properties of the tested samples (IC_50_ (mg /mL)).

Samples	DPPH	ABTS	CUPRAC	FRAP	PBD	Chelating
Early somatic embryos	2.13 ± 0.11 ^c^	1.59 ± 0.02 ^c^	1.63 ± 0.01 ^c^	1.03 ± 0.01 ^c^	2.13 ± 0.04 ^c^	1.93 ± 0.10 ^b^
Mature somatic embryos	2.41 ± 0.06 ^d^	1.68 ± 0.01 ^d^	1.72 ± 0.02 ^d^	1.01 ± 0.01 ^c^	2.22 ± 0.22 ^cd^	>3
Germinated somatic embryos	0.62 ± 0.01 ^b^	0.83 ± 0.01 ^b^	0.92 ± 0.01 ^b^	0.55 ± 0.01 ^b^	1.87 ± 0.05 ^b^	2.04 ± 0.03 ^b^
Trolox	0.06 ± 0.01 ^a^	0.09 ± 0.01 ^a^	0.11 ± 0.01 ^a^	0.04 ± 0.01 ^a^	0.52 ± 0.02 ^a^	nt
EDTA	nt	nt	nt	nt	nt	0.02 ± 0.001 ^a^

Values are expressed as mean ± S.D. nt: no tested. PBD: Phosphomolybdenum. Different letters indicate significant differences in the tested samples (*p* < 0.05).

**Table 6:** Enzyme inhibitory effects of the tested samples (IC50 (mg /mL)).

Samples	AChE	BChE	Tyrosinase	Amylase
Early somatic embryos	0.83 ± 0.05 ^bc^	1.59 ± 0.05 ^c^	0.76 ± 0.01 ^bc^	1.32 ± 0.02 ^b^
Mature somatic embryos	0.75 ± 0.02 ^b^	1.28 ± 0.13 ^b^	0.79 ± 0.02 ^c^	1.51 ± 0.01 ^d^
Germinated somatic embryos	0.83 ± 0.02 ^c^	1.27 ± 0.23 ^b^	0.73 ± 0.02 ^b^	1.39 ± 0.03 ^c^
Galantamine	0.003 ± 0.001 ^a^	0.007 ± 0.002 ^a^	nt	nt
Kojic acid	nt	nt	0.08 ± 0.001 ^a^	nt
Acarbose	nt	nt	nt	0.68 ± 0.01 ^a^

Values are expressed as mean ± S.D. nt: no tested. Different letters indicate significant differences in the tested samples (*p* < 0.05).

## Data Availability

The data presented in the present study are available in the article.

## Supplementary Materials

The following supporting information can be downloaded at: https://www.mdpi.com/article/10.3390/plants12051065/s1, Table S1: Chemical composition of early somatic embryo extract; Table S2: Chemical composition of mature; somatic embryo extract Table S3: Chemical composition of germinated somatic embryo extract; Figure S1: Total ion chromatogram of early somatic embryos extract of *C. orbiculata* in positive (a) and negative (b) mode; Figure S2: Total ion chromatogram of mature somatic embryos extract of *C. orbiculata* in positive (a) and negative (b) mode; Figure S3: Total ion chromatogram of germinated somatic embryos extract of *C. orbiculata* in positive (a) and negative (b) mode.
