# Supplementary material for: Phytochemical Composition and Biological Activities of Extracts from Early, Mature, and Germinated Somatic Embryos of Cotyledon orbiculata L
Source: Plants (Basel). 2023 Feb 27;12(5):1065. doi: 10.3390/plants12051065 (PMC10005620; doi:10.3390/plants12051065)
Supplement: Supplementary file 1 [file plants-12-01065-s001.zip › plants-2105421-supplementary.pdf]

Article

Phytochemical composition and biological activities of extracts from early, mature, and germinated somatic embryos of *Cotyledon orbiculata* L.

Gokhan Zengin [1], Zoltán Cziáky [2], József Jekő [2], Kyung Won Kang [3], José Manuel Lorenzo [4,5], and Iyyakkannu Sivanesan [6,*]

[1] Department of Biology, Faculty of Science, Selcuk University, 42250 Konya, Turkey
[2] Agricultural and Molecular Research and Service Institute, University of Nyíregyháza, Nyíregyháza, Hungary
[3] Babo Orchid Farm, Gyeonggi-do, 472-831 Namyangju-si, South Korea
[4] Centro Tecnológico de la Carne de Galicia, Rúa Galicia Nº 4, Parque Tecnológico de Galicia, San Cibrao dasViñas, 32900 Ourense, Spain
[5] Facultade de Ciencias, Universidade de Vigo,Área de Tecnoloxía dos Alimentos, 32004 Ourense, Spain
[6] Department of Bioresources and Food Science, Institute of Natural Science and Agriculture, Konkuk University, 05029 Seoul, South Korea
* Correspondence: isivanesan@gmail.com or siva74@konkuk.ac.kr; Tel.: +82-2450-0576

Table S1. Chemical composition of early somatic embryo extract.

No.	Name	Formula	Rt	$[M+H]^+$	$[M-H]^-$	Fra 1	Fra 2	Fra 3	Fra 4	Fra 5
1	Trigonelline	$C_7H_7NO_2$	1.27	138.05550		110.0603	96.0450	94.0656	92.0499	65.0391
2	Nicotinic acid (Niacin)	$C_6H_5NO_2$	1.49	124.03986		96.0449	80.0501	78.0345		
3	Nicotinamide	$C_6H_6N_2O$	1.61	123.05584		106.0289	96.0449	80.0501		
4[1]	Gallic acid (3,4,5-Trihydroxybenzoic acid)	$C_7H_6O_5$	2.73		169.01370	125.0225	97.0281	81.0330	69.0330	
5	Phenethylamine	$C_8H_{11}N$	4.30	122.09698		105.0702	103.0545	79.0548		
6	Dihydroxybenzoic acid	$C_7H_6O_4$	10.55		153.01879	123.0073	109.0280	108.0202	95.0123	85.0279
7	Caffeic acid	$C_9H_8O_4$	15.25		179.03444	135.0438	107.0487			
8[1]	Taxifolin (Dihydroquercetin)	$C_{15}H_{12}O_7$	19.95		303.05048	285.0404	177.0181	175.0385	153.0182	125.0229
9	cis-3-[(4-hydroxy-3-methoxyphenyl)-prop-2-enoyl]oxybutanedioic acid	$C_{14}H_{14}O_8$	20.64	311.07670		177.0545	149.0597	145.0284	117.0337	89.0390
10	Eriodictyol-O-hexoside	$C_{21}H_{22}O_{11}$	20.85		449.10839	287.0562	151.0023	135.0438	107.0123	
11	trans-3-[(4-hydroxy-3-methoxyphenyl) prop-2-enoyl]oxybutanedioic acid	$C_{14}H_{14}O_8$	21.50	311.07670		177.0546	149.0598	145.0284	117.0337	89.0390
12	Luteolin-O-hexoside isomer 1	$C_{21}H_{20}O_{11}$	22.45		447.09274	327.0519	285.0405	284.0328	256.0368	151.0023
13	Luteolin-O-hexoside isomer 2	$C_{21}H_{20}O_{11}$	22.92		447.09274	327.0511	285.0405	284.0327	256.0369	151.0026
14	Hyperoside (Quercetin-3-O-galactoside)	$C_{21}H_{20}O_{12}$	23.26		463.08765	301.0362	300.0276	271.0248	255.0297	178.9977
15[1]	Isoquercitrin (Quercetin-3-O-glucoside)	$C_{21}H_{20}O_{12}$	23.50		463.08765	301.0356	300.0275	271.0248	255.0294	178.9975
16[1]	Eriodictyol (3′,4′,5,7-Tetrahydroxyflavanone)	$C_{15}H_{12}O_6$	25.46		287.05556	269.0453	151.0023	135.0438	125.0228	107.0123
17[1]	Quercetin (3,3',4',5,7-Pentahydroxyflavone)	$C_{15}H_{10}O_7$	27.57		301.03483	273.0403	178.9976	151.0024	121.0280	107.0124
18[1]	Naringenin (4',5,7-Trihydroxyflavanone)	$C_{15}H_{12}O_5$	27.77		271.06065	227.0704	177.0183	151.0024	119.0487	107.0123
19[1]	Luteolin (3',4',5,7-Tetrahydroxyflavone)	$C_{15}H_{10}O_6$	28.46		285.03991	217.0500	199.0392	175.0387	151.0023	133.0281
20[1]	Apigenin (4',5,7-Trihydroxyflavone)	$C_{15}H_{10}O_5$	30.31		269.04500	225.0551	201.0556	151.0024	149.0232	117.0330
21	Chrysoeriol (3'-Methoxy-4',5,7-trihydroxyflavone)	$C_{16}H_{12}O_6$	30.53		299.05556	284.0327	256.0374	227.0348	151.0023	107.0124
22	Trihydroxy-trimethoxy(iso)flavone isomer I	$C_{18}H_{16}O_8$	32.95	361.09235		346.0680	345.0600	331.0444	317.0660	303.0492
23	Dihydroxy-dimethoxy(iso)flavone	$C_{17}H_{14}O_6$	32.98	315.08686		300.0628	299.0549	271.0600	257.0443	
24	Chrysin (5,7-Dihydroxyflavone)	$C_{15}H_{10}O_4$	33.86	255.06573		209.0600	153.0182	129.0337	103.0546	67.0185
25	Dimethoxy(iso)flavone	$C_{17}H_{14}O_4$	34.16	283.09704		268.0728	267.0647	239.0700	225.0533	
26[1]	Galangin (3,5,7-Trihydroxyflavone)	$C_{15}H_{10}O_5$	34.74	271.06065		215.0697	197.0589	165.0148	153.0181	105.0338
27	Trimethoxy(iso)flavone	$C_{18}H_{16}O_5$	35.01	313.10760		298.0837	297.0757	280.0729	279.0652	252.0781
28	Dihydroxy-methoxy(iso)flavone	$C_{16}H_{12}O_5$	35.11	285.07630		270.0521	269.0446	242.0572	167.0339	119.0493
29	Hydroxy-trimethoxy(iso)flavone	$C_{18}H_{16}O_6$	36.92	329.10251		314.0781	313.0708	299.0548	271.0599	
30	Hydroxy-methoxy(iso)flavone	$C_{16}H_{12}O_4$	38.10	269.08138		254.0572	226.0622	167.0339		
31	Linoleamide	$C_{18}H_{33}NO$	44.47	280.26404		263.2369	245.2264	109.1015	95.0860	81.0704

32	Oleamide	$C_{18}H_{35}NO$	45.76	282.27969		265.2526	247.2420	135.1171	83.0861	69.0705

[1]Confirmed by the standard.

Table S2. Chemical composition of mature somatic embryo extract.

No.	Name	Formula	Rt	$[M+H]^+$	$[M-H]^-$	Fra1	Fra 2	Fra 3	Fra 4	Fra 5
1	Trigonelline	$C_7H_7NO_2$	1.26	138.05550		110.0603	96.0450	94.0656	92.0499	65.0392
2	Nicotinic acid (Niacin)	$C_6H_5NO_2$	1.54	124.03986		96.0449	80.0501	78.0345		
3	Nicotinamide	$C_6H_6N_2O$	1.63	123.05584		106.0290	96.0449	80.0500		
6	Dihydroxybenzoic acid	$C_7H_6O_4$	10.41		153.01879	123.0073	109.0279	108.0201	95.0123	85.0278
7	Caffeic acid	$C_9H_8O_4$	15.17		179.03444	135.0438	107.0489			
8[1]	Taxifolin (Dihydroquercetin)	$C_{15}H_{12}O_7$	19.90		303.05048	285.0403	177.0177	175.0384	153.0183	125.0228
10	Eriodictyol-O-hexoside	$C_{21}H_{22}O_{11}$	20.79		449.10839	287.0563	151.0024	135.0438	107.0123	
9	cis-3-[(4-hydroxy-3-Methoxyphenyl) prop-2-enoyl]oxybutanedioic acid	$C_{14}H_{14}O_8$	20.90	311.07670		177.0546	149.0597	145.0284	117.0337	89.0390
11	trans-3-[(4-hydroxy-3-Methoxyphenyl) prop-2-enoyl]oxybutanedioic acid	$C_{14}H_{14}O_8$	21.45	311.07670		177.0546	149.0597	145.0284	117.0337	89.0390
33	Quercetin-O-pentosylhexoside	$C_{26}H_{28}O_{16}$	21.56		595.12991	301.0354	300.0275	271.0247	255.0296	178.9975
12	Luteolin-O-hexoside isomer 1	$C_{21}H_{20}O_{11}$	22.35		447.09274	327.0499	285.0404	284.0327	256.0367	151.0021
13	Luteolin-O-hexoside isomer 2	$C_{21}H_{20}O_{11}$	22.83		447.09274	327.0499	285.0405	284.0328	256.0369	151.0024
14	Hyperoside (Quercetin-3-O-galactoside)	$C_{21}H_{20}O_{12}$	23.23		463.08765	301.0349	300.0275	271.0248	255.0298	178.9971
15[1]	Isoquercitrin (Quercetin-3-O-glucoside)	$C_{21}H_{20}O_{12}$	23.43		463.08765	301.0360	300.0275	271.0248	255.0296	178.9975
16[1]	Eriodictyol (3′,4′,5,7-Tetrahydroxyflavanone)	$C_{15}H_{12}O_6$	25.42		287.05556	269.0461	151.0024	135.0438	125.0229	107.0123
17[1]	Quercetin (3,3',4',5,7-Pentahydroxyflavone)	$C_{15}H_{10}O_7$	27.55		301.03483	273.0412	178.9975	151.0024	121.0280	107.0124
18[1]	Naringenin (4',5,7-Trihydroxyflavanone)	$C_{15}H_{12}O_5$	27.75		271.06065	227.0709	177.0181	151.0023	119.0487	107.0123
19[1]	Luteolin (3',4',5,7-Tetrahydroxyflavone)	$C_{15}H_{10}O_6$	28.42		285.03991	217.0499	199.0393	175.0390	151.0024	133.0281
20[1]	Apigenin (4',5,7-Trihydroxyflavone)	$C_{15}H_{10}O_5$	30.27		269.04500	225.0554	201.0545	151.0020	149.0229	117.0330
21	Chrysoeriol (3'-Methoxy-4',5,7-trihydroxyflavone)	$C_{16}H_{12}O_6$	30.49		299.05556	284.0328	256.0371	227.0366	151.0020	107.0124
34	Dihydroxy-trimethoxy(iso)flavone isomer 1	$C_{17}H_{14}O_7$	31.06		329.06613	314.0436	313.0363	299.0196	271.0252	227.0345
22	Trihydroxy-trimethoxy(iso)flavone isomer I	$C_{18}H_{16}O_8$	32.93	361.09235		346.0685	345.0616	331.0439	317.0653	303.0499
23	Dihydroxy-dimethoxy(iso)flavone	$C_{17}H_{14}O_6$	32.96	315.08686		300.0629	299.0549	271.0602	257.0445	
35	Dihydroxy-trimethoxy(iso)flavone isomer 2	$C_{17}H_{14}O_7$	33.34		329.06613	314.0435	299.0197	271.0246		
24	Chrysin (5,7-Dihydroxyflavone)	$C_{15}H_{10}O_4$	33.84	255.06573		209.0597	153.0183	129.0337	103.0547	67.0186
25	Dimethoxy(iso)flavone	$C_{17}H_{14}O_4$	34.13	283.09704		268.0729	267.0644	239.0701	225.0542	
26[1]	Galangin (3,5,7-Trihydroxyflavone)	$C_{15}H_{10}O_5$	34.72	271.06065		215.0700	197.0598	165.0148	153.0180	105.0338
27	Trimethoxy(iso)flavone	$C_{18}H_{16}O_5$	34.97	313.10760		298.0837	297.0757	280.0730	279.0650	252.0779
28	Dihydroxy-methoxy(iso)flavone	$C_{16}H_{12}O_5$	35.09	285.07630		270.0520	269.0440	242.0573	167.0341	119.0492

29	Hydroxy-trimethoxy(iso)flavone	$C_{18}H_{16}O_6$	36.89	329.10251		314.0781	313.0704	299.0548	271.0599	
30	Hydroxy-methoxy(iso)flavone	$C_{16}H_{12}O_4$	38.07	269.08138		254.0574	226.0624	167.0341		
31	Linoleamide	$C_{18}H_{33}NO$	44.45	280.26404		263.2369	245.2265	109.1016	95.0860	81.0705
32	Oleamide	$C_{18}H_{35}NO$	45.71	282.27969		265.2528	247.2419	135.1169	83.0861	69.0705

[1]Confirmed by the standard.

Table S3. Chemical composition of germinated somatic embryo extract.

No.	Name	Formula	Rt	$[M+H]^+$	$[M-H]^-$	Fra 1	Fra 2	Fra 3	Fra 4	Fra 5
1	Trigonelline	$C_7H_7NO_2$	1.26	138.05550		110.0604	96.0449	94.0656	92.0499	65.0392
2	Nicotinic acid (Niacin)	$C_6H_5NO_2$	1.48	124.03986		96.0449	80.0501	78.0345		
3	Nicotinamide	$C_6H_6N_2O$	1.61	123.05584		106.0291	96.0449	80.0501		
4[1]	Gallic acid (3,4,5-Trihydroxybenzoic acid)	$C_7H_6O_5$	2.68		169.01370	125.0230	97.0278	81.0330	69.0329	
6	Dihydroxybenzoic acid	$C_7H_6O_4$	10.43		153.01879	123.0073	109.0280	108.0202	95.0123	85.0279
7	Caffeic acid	$C_9H_8O_4$	15.18		179.03444	135.0438	107.0488			
8[1]	Taxifolin (Dihydroquercetin)	$C_{15}H_{12}O_7$	19.92		303.05048	285.0404	177.0183	175.0391	153.0179	125.0229
9	cis-3-[(4-hydroxy-3-Methoxyphenyl)-prop-2-enoyl]oxybutanedioic acid	$C_{14}H_{14}O_8$	20.78	311.07670		177.0546	149.0597	145.0284	117.0337	89.0391
10	Eriodictyol-O-hexoside	$C_{21}H_{22}O_{11}$	20.85		449.10839	287.0562	151.0024	135.0438	107.0123	
11	trans-3-[(4-hydroxy-3-methoxyphenyl) prop-2-enoyl]oxybutanedioic acid	$C_{14}H_{14}O_8$	21.45	311.07670		177.0546	149.0598	145.0284	117.0337	89.0390
12	Luteolin-O-hexoside isomer 1	$C_{21}H_{20}O_{11}$	22.39		447.09274	327.0509	285.0405	284.0331	256.0380	151.0024
14	Hyperoside (Quercetin-3-O-galactoside)	$C_{21}H_{20}O_{12}$	23.22		463.08765	301.0361	300.0277	271.0248	255.0298	178.9975
15[1]	Isoquercitrin (Quercetin-3-O-glucoside)	$C_{21}H_{20}O_{12}$	23.44		463.08765	301.0359	300.0277	271.0248	255.0297	178.9975
16[1]	Eriodictyol (3′,4′,5,7-Tetrahydroxyflavanone)	$C_{15}H_{12}O_6$	25.43		287.05556	269.0454	151.0024	135.0439	125.0230	107.0124
17[1]	Quercetin (3,3',4',5,7-Pentahydroxyflavone)	$C_{15}H_{10}O_7$	27.55		301.03483	273.0410	178.9976	151.0024	121.0280	107.0124
18[1]	Naringenin (4',5,7-Trihydroxyflavanone)	$C_{15}H_{12}O_5$	27.76		271.06065	227.0712	177.0183	151.0024	119.0488	107.0124
19[1]	Luteolin (3',4',5,7-Tetrahydroxyflavone)	$C_{15}H_{10}O_6$	28.42		285.03991	217.0497	199.0391	175.0388	151.0024	133.0282
20[1]	Apigenin (4',5,7-Trihydroxyflavone)	$C_{15}H_{10}O_5$	30.26		269.04500	225.0552	201.0544	151.0025	149.0232	117.0331
36[1]	Isorhamnetin (3'-Methoxy-3,4',5,7-tetrahydroxyflavone)	$C_{16}H_{12}O_7$	30.41		315.05048	300.0276	283.0247	271.0249	164.0105	151.0026
22	Trihydroxy-trimethoxy(iso)flavone isomer I	$C_{18}H_{16}O_8$	31.35	361.09235		346.0681	345.0606	331.0447	317.0661	303.0498
37	Rhamnetin (7-Methoxy-3,3',4',5-tetrahydroxyflavone)	$C_{16}H_{12}O_7$	32.41		315.05048	300.0273	193.0135	165.0181	121.0281	97.0280
38	Trihydroxy-trimethoxy(iso)flavone isomer II	$C_{18}H_{16}O_8$	32.94	361.09235		346.0680	345.0609	331.0452	317.0654	303.0490
23	Dihydroxy-dimethoxy(iso)flavone	$C_{17}H_{14}O_6$	32.96	315.08686		300.0628	299.0549	271.0601	257.0446	
24	Chrysin (5,7-Dihydroxyflavone)	$C_{15}H_{10}O_4$	33.84	255.06573		209.0594	153.0183	129.0335	103.0546	67.0184
25	Dimethoxy(iso)flavone	$C_{17}H_{14}O_4$	34.13	283.09704		268.0729	267.0653	239.0701	225.0549	
26[1]	Galangin (3,5,7-Trihydroxyflavone)	$C_{15}H_{10}O_5$	34.72	271.06065		215.0700	197.0596	165.0148	153.0181	105.0338

27	Trimethoxy(iso)flavone	$C_{18}H_{16}O_5$	34.99	313.10760		298.0837	297.0757	280.0729	279.0647	252.0779
28	Dihydroxy-methoxy(iso)flavone	$C_{16}H_{12}O_5$	35.05	285.07630		270.0523	269.0445	242.0573	167.0340	119.0492
29	Hydroxy-trimethoxy(iso)flavone	$C_{18}H_{16}O_6$	36.90	329.10251		314.0785	313.0693	299.0549	271.0601	
30	Hydroxy-methoxy(iso)flavone	$C_{16}H_{12}O_4$	38.07	269.08138		254.0573	226.0623	167.0338		
31	Linoleamide	$C_{18}H_{33}NO$	44.45	280.26404		263.2369	245.2262	109.1015	95.0860	81.0705
32	Oleamide	$C_{18}H_{35}NO$	45.74	282.27969		265.2528	247.2417	135.1168	83.0861	69.0705

[1]Confirmed by the standard.

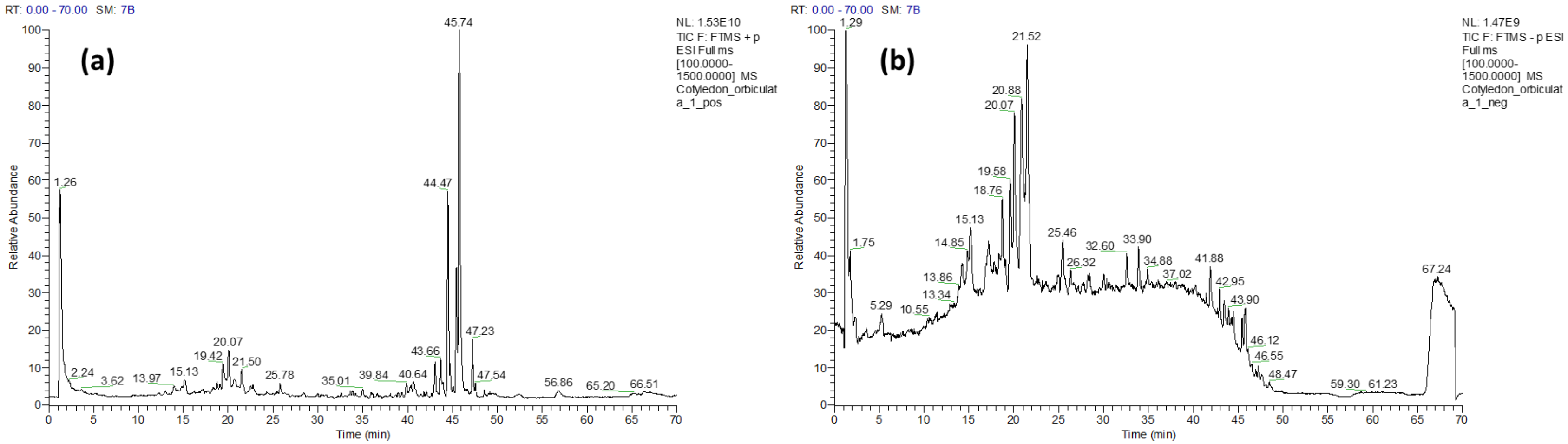

Figure S1. Total ion chromatogram of early somatic embryos extract of *C. orbiculata* in positive (a) and negative (b) mode.

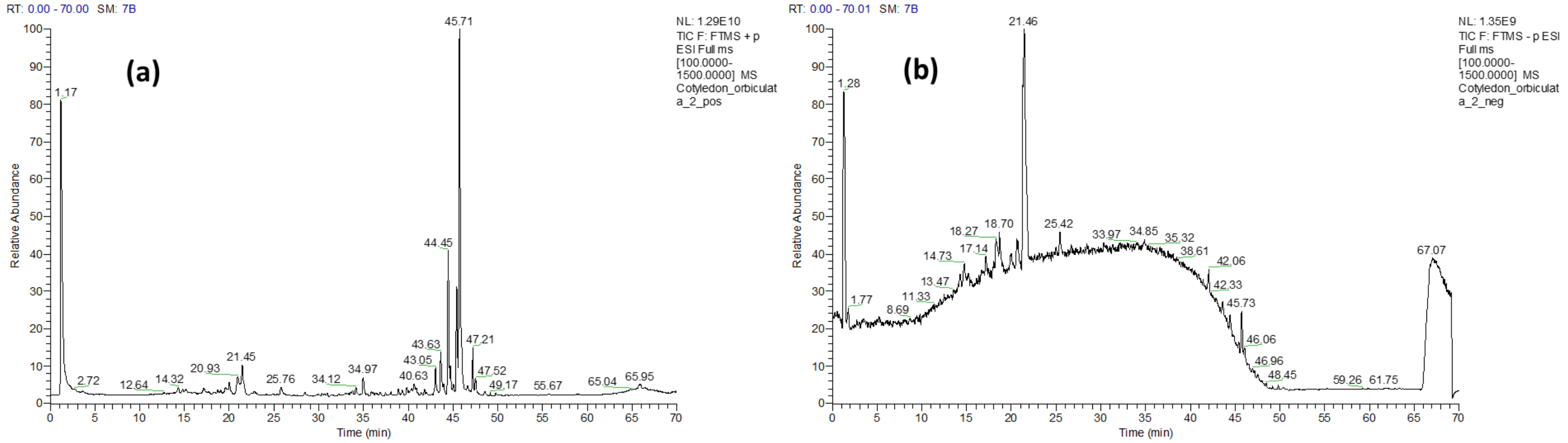

Figure S2. Total ion chromatogram of mature somatic embryos extract of *C. orbiculata* in positive (a) and negative (b) mode.

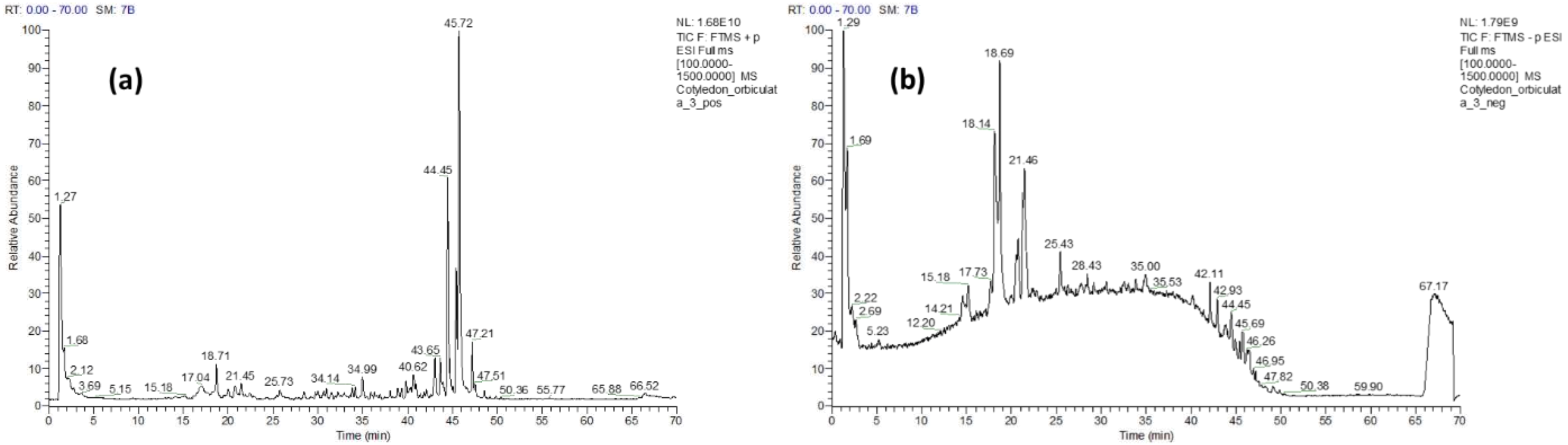

Figure S3. Total ion chromatogram of germinated somatic embryos extract of *C. orbiculata* in positive (a) and negative (b) mode.
